# The Clathrin adaptor AP-1 and Stratum act in parallel pathways to control Notch activation in *Drosophila* sensory organ precursors cells

**DOI:** 10.1242/dev.191437

**Published:** 2021-01-11

**Authors:** Karen Bellec, Mathieu Pinot, Isabelle Gicquel, Roland Le Borgne

**Affiliations:** Université Rennes, CNRS, IGDR (Institut de Génétique et Développement de Rennes) - UMR 6290, F-35000 Rennes, France

**Keywords:** Notch signalling, Intracellular trafficking, Asymmetric cell division, Stratum, AP-1

## Abstract

*Drosophila* sensory organ precursors divide asymmetrically to generate pIIa/pIIb cells, the identity of which relies on activation of Notch at cytokinesis. Although Notch is present apically and basally relative to the midbody at the pIIa-pIIb interface, the basal pool of Notch is reported to be the main contributor for Notch activation in the pIIa cell. Intra-lineage signalling requires appropriate apico-basal targeting of Notch, its ligand Delta and its trafficking partner Sanpodo. We have previously reported that AP-1 and Stratum regulate the trafficking of Notch and Sanpodo from the *trans*-Golgi network to the basolateral membrane. Loss of AP-1 or Stratum caused mild *Notch* gain-of-function phenotypes. Here, we report that their concomitant loss results in a penetrant *Notch* gain-of-function phenotype, indicating that they control parallel pathways. Although unequal partitioning of cell fate determinants and cell polarity were unaffected, we observed increased amounts of signalling-competent Notch as well as Delta and Sanpodo at the apical pIIa-pIIb interface, at the expense of the basal pool of Notch. We propose that AP-1 and Stratum operate in parallel pathways to localize Notch and control where receptor activation takes place.

## Abstract (precis)

**Summary:** The Notch pathway activation relies on the correct localization of the Notch signalling actors. We report that AP-1 and Stratum ensure the basolateral targeting of Notch during asymmetric cell division.

## INTRODUCTION

Cell-cell signalling by the evolutionarily conserved Notch receptor promotes cell fate acquisition in a large variety of developmental processes in metazoans (Artavanis-Tsakonas et al., 1999; Bray, 1998; Kopan and Ilagan, 2009). In most cases, the Notch receptor is activated by transmembrane ligands present at the plasma membrane of adjacent cells. Following binding to Notch, endocytosis of the ligand induces pulling forces that drive a change in the conformation of the Notch extracellular domain, thereby unmasking the S2 cleavage site of Notch (Gordon et al., 2015; Langridge and Struhl, 2017; Meloty-Kapella et al., 2012; Seo et al., 2016; Shergill et al., 2012; Wang and Ha, 2013). This regulated cleavage is followed by a constitutive proteolytic cleavage of Notch by the gamma secretase complex (Mumm et al., 2000; Struhl and Adachi, 2000), giving rise to the Notch intracellular domain (NICD), a polypeptide that translocates into the nucleus to act as a transcriptional co-activator (Artavanis-Tsakonas et al., 1999; Bray, 1998; Kopan and Ilagan, 2009). As proteolytic activation of the Notch receptor is irreversible, Notch activation needs to be tightly controlled in time and in space. The model system of asymmetric cell division of the sensory organ precursors (SOPs) in the pupal notum of *Drosophila* has been instrumental in identifying the site of Notch activation at the cell surface. SOPs are polarized epithelial cells that divide asymmetrically within the plane of the epithelium to generate two daughter cells, the fate of which depends on the differential activation of Notch signalling (Schweisguth, 2015). The differential activation of Notch relies on the unequal partitioning of the two cell fate determinants Neuralized (Neur) and Numb in the anterior SOP daughter cell (Le Borgne and Schweisguth, 2003; Rhyu et al., 1994). Neur promotes the endocytosis of Delta, one of the Notch ligands (Le Borgne and Schweisguth, 2003), while Numb inhibits the recycling of Notch and its co-factor Sanpodo (Spdo) towards the plasma membrane – promoting their targeting towards late endosomal compartments instead (Cotton et al., 2013; Couturier et al., 2013; Johnson et al., 2016; Upadhyay et al., 2013). Consequently, the anterior cell adopts the pIIb identity while Notch is selectively activated in the posterior cell that adopts the pIIa fate. Combination of live-imaging, FRAP experiments using NiGFP and photo-tracking of photoconverted NimMaple3 has revealed that proteolytic activation of Notch occurs during SOP cytokinesis and that a specific pool of Notch receptors located basal to the midbody is the main contributor to the signalling in the pIIa cell (Trylinski et al., 2017). These data suggest a polarized trafficking of Notch, Delta and Spdo towards this specific subcellular location during cytokinesis.

**Fig. 1.**
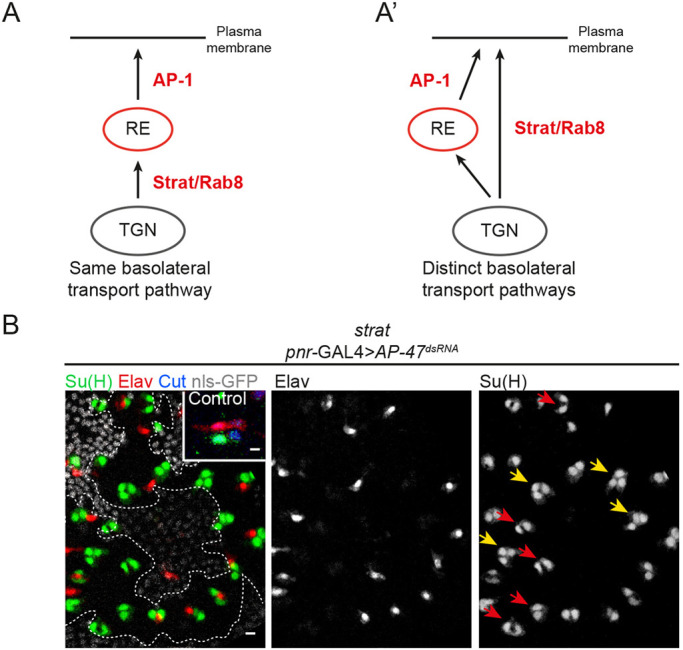
**Loss of Strat and AP-1 causes *Notch* gain-of-function phenotype within the SO lineage.** (A,A′) Schematic representations of the involvement of AP-1 and Strat in the same (A) or in distinct (A′) basolateral transport pathway. (B) Projection of confocal sections of a pupal notum at 24 h APF in a wild-type organ or in *strat* SOPs expressing *pnr*-GAL4>*AP-47^dsRNA^*. *strat* mutant cells were identified by the absence of the nuclear marker nls-GFP (grey). Sockets were identified with Su(H) [anti-Su(H), green] and neurons with Elav (anti-Elav, red). In the wild-type organ, cells composing the SO are identified with Cut (anti-Cut, blue). Scale bars: 5 μm. Yellow and red arrows show transformed SOs containing three or four Su(H)-positive cells or two Su(H)-positive cells without neurons, respectively. Inset shows a control SO lineage.

We have previously reported that the clathrin adaptor complex AP-1 regulates the polarized sorting of Notch and Spdo from the *trans*-Golgi network (TGN) and the recycling endosomes (RE) towards the plasma membrane (Benhra et al., 2011). Loss of AP-1 causes stabilization of Notch and Spdo at the adherens junctions following SOP division, a phenotype associated with a mild *Notch* gain-of-function phenotype (GOF). Unequally partitioned Numb controls the endosomal sorting of Notch/Spdo after asymmetric division and prevents their recycling to the plasma membrane (Couturier et al., 2013). This recycling event relies on AP-1 activity (Cotton et al., 2013). We also reported that Stratum (Strat), a chaperone regulating Rab8 recruitment, controls the exit from the Golgi apparatus, as well as the basolateral targeting of Notch, Delta and Spdo (Bellec et al., 2018). As for AP-1, loss of Strat leads to an enrichment in Notch and Spdo at the apical pole of SOP daughter cells associated with a mild *Notch* gain-of-function phenotype. Because AP-1 and Strat/Rab8 both regulate Notch and Spdo trafficking to the basolateral plasma membrane, a possible interpretation of our data is that AP-1 and Strat act in the same transport pathway and therefore are simply fine-tuning the regulation of Notch-Delta trafficking and activation. An alternative explanation could be that AP-1 and Strat function in two parallel pathways (Fig. 1A,A′) to ensure proper basal localization of the Notch receptor. In this scenario, loss of one of the two components could be at least in part compensated by the other. A prediction of this second hypothesis is that the concomitant loss of AP-1 and Strat would exhibit a stronger phenotype.

In this study, we have investigated the consequences of simultaneous disruption of Strat and AP-1 function. We report that concomitant impairment of Strat and AP-1 impacts neither the overall apico-basal polarity of epithelial cells nor the unequal partitioning of Numb and Neur at SOP cytokinesis. However, it does result in increased amounts of Notch, Spdo and Delta at the apical pole of the SOP daughter cells, whereas Notch and Spdo, normally localized basally at the pIIa-pIIb interface (Trylinski et al., 2017), are drastically reduced. This phenotype is associated with a pIIb-to-pIIa cell fate transformation that is much more penetrant than that of the single *AP-1* or *strat* mutants alone. Photoconversion and spatio-temporal monitoring of NimMaple3 in the context of simultaneous impairment of AP-1 and Strat indicate that this fate conversion is a consequence of aberrant Notch localization. Upon simultaneous loss of AP-1 and Strat, Notch receptors localized in excess at the apical pole are proteolytically activated and the resulting NICD is translocated to nuclei of both SOP daughter cells. This would explain the *Notch* gain-of-function phenotype as Notch activation may now occur in both SOP daughter cells. We propose a model according which AP-1 and Strat control two parallel transport routes that both contribute to the polarized transport of Notch and Spdo to the basal pIIa-pIIb interface to ensure binary cell fate acquisition at SOP cytokinesis.

## RESULTS

### Simultaneous loss of AP-1 and Strat causes a penetrant *Notch* GOF phenotype

To test whether AP-1 and Strat are involved in the same pathway to regulate the activation of the Notch signalling pathway, we induced clones of cells homozygous mutant for a null mutation of *strat* generated by CRISPR (Bellec et al., 2018) in which the µ subunit of the AP-1 complex (also known as AP-47) was silenced using previously characterized tools (Benhra et al., 2011). Silencing of *AP-47* is hereafter referred to as loss of AP-1. If Strat and AP-1 act in two different basolateral pathways, we expect the *Notch* gain-of-function phenotype observed in absence of the two proteins to be much more penetrant. In the wild-type sensory organ (SO), the pIIb cell divides twice to generate the internal cells, among which is one Elav-positive neuron. The pIIa cell divides once to generate the external cells, among which is one socket cell identified by Suppressor of Hairless [Su(H)]. Su(H) can therefore be used as a read-out of cell fate transformations to monitor the effect of individual or simultaneous impairment of Strat and AP-1 (Fig. 1). In agreement with previous studies, mild *Notch* gain-of-function phenotypes revealed by an excess of Su(H)-positive socket cells were observed in 6.7% and 7.5% of SOs mutant for *strat* or depleted of AP-1, respectively (Bellec et al., 2018; Benhra et al., 2011). However, simultaneous loss of AP-1 and Strat led to a much stronger *Notch* gain-of-function phenotype (49% of transformed SOs, Fig. 1B, Table 1). Among them, 34% have precisely two Su(H)-positive and 49% have at least two Su(H)-positive cells with no Elav-positive cells, indicating that these SOs were already transformed at the two-cell stage (Table 1). These results were confirmed using a different method, i.e. silencing both AP-1 and Strat using dsRNA (Table 1). The strong enhancement of the *Notch* gain-of-function phenotypes in this lineage analysis indicates that AP-1 and Strat act in distinct and complementary pathways to regulate Notch activation.

**Table 1.**
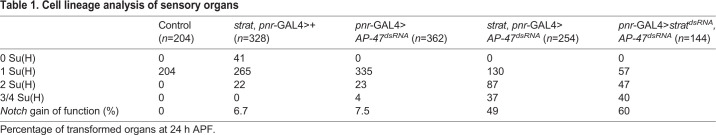
**Cell lineage analysis of sensory organs**

### Unequal partitioning of cell-fate determinants is unaffected by the loss of AP-1 and Strat

**Fig. 2.**
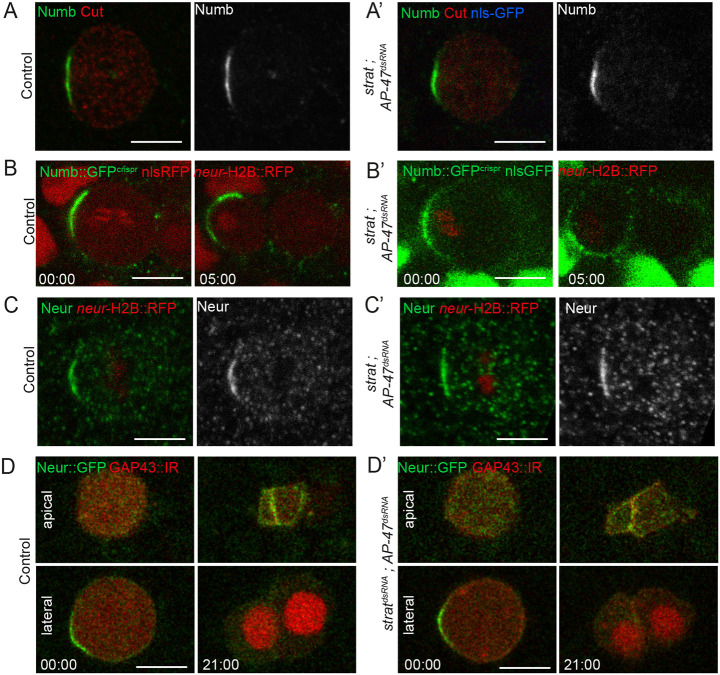
**Loss of Strat and AP-1 does not affect the localization of Numb and Neur.** (A,A′) Localization of Numb (anti-Numb, green) in wild-type SOP (*n*=22 of prophase/prometaphase and dividing SOPs) and in *strat* SOP expressing *pnr*-GAL4>*AP-47^dsRNA^* (*n*=16 of prophase/prometaphase and dividing SOPs). SOPs and SOP daughter cells were identified with Cut (anti-Cut, red). (B,B′) Time-lapse imaging of Numb::GFP^crispr^ (green) in dividing wild-type SOPs expressing Histone2B::RFP under the *neur* promoter (red; B, *n*=10) and in dividing *strat* SOPs expressing Histone2B::RFP under the *neur* promoter (red) and *pnr*-GAL4>*AP-47^dsRNA^* (B′, *n*=15). (C,C′) Localization of Neur (anti-Neur, green) in wild-type SOPs (*n*=14 of prophase/prometaphase and dividing SOPs) and in *strat* SOPs expressing *pnr*-GAL4>*AP-47^dsRNA^* (*n*=7 of prophase/prometaphase and dividing SOPs). SOPs and SOP daughter cells were identified with Histone2B::RFP expressed under the *neur* promoter (red). A similar phenotype was observed in the *pnr*-GAL4>*strat^dsRNA^, AP-47^dsRNA^* (data not shown, *n*=8 of prophase/prometaphase and dividing SOPs). (D,D′) Time-lapse imaging of Neur::GFP (Bac Rescue, green) during division of control (D, *n*=8) or *pnr*-GAL4> *strat^dsRNA^; AP-47^dsRNA^* (D′, *n*=13) SOPs expressing GAP43::iRFP together iRFP::nls (red) under the control of the *neur* promoter. Neur::GFP localized at the apical interface of SOP daughter cells at t21 min (D,D′). Time is in min:s and the time 00:00 corresponds to the onset of anaphase in SOPs. Scale bar: 5 μm.

As Strat and AP-1 regulate basolateral trafficking, we first monitored the effect of loss of these two regulators on the distribution of several cell polarity markers. We noticed that, as in *AP-1* mutants, the cuticle is thinner and less pigmented than in controls (Fig. S1A-B′). Despite these cuticle defects, the localization of the junctional markers DE-Cadherin, Par3 and Coracle is unaffected by the loss of AP-1 and Strat, suggesting that cell polarity is not altered (Fig. S1C,D). However, time-lapse imaging revealed that Par3, instead of being enriched at the posterior pole of SOP prior to mitosis (Bellaïche et al., 2001; Besson et al., 2015), distributes uniformly at the apical cortex of SOP upon loss of Strat and AP-1 at this stage of the cell cycle (orange arrows, Fig. S1D). This defect in planar cell polarity (PCP) regulation is likely caused by the loss of AP-1, as reported in the *Drosophila* wing (Carvajal-Gonzalez et al., 2012). Despite this defect, during mitosis, Par3 localizes normally at the posterior cortex upon loss of AP-1 and Strat, as is the case in control SOP (Fig. S2A,B). As Par3 is required for the asymmetric localization of Numb and Neur during mitosis (Bellaïche et al., 2001; Langevin et al., 2005), we anticipated that their localization would not be affected in *strat* mutant SOPs depleted of AP-1. Indeed, we found that Numb and Neur localize asymmetrically during prometaphase in the absence of Strat and AP-1 (Fig. 2A,A′,C,C′). Live-imaging of Numb::GFP^crispr^ (Bellec et al., 2018) and Neur::GFP (Perez-Mockus et al., 2017) revealed that they are unequally partitioned in the anterior SOP daughter cell, in a similar manner to the control situation (Fig. 2B,B′,D,D′). We conclude that the *Notch* gain-of-function phenotype is unlikely to be caused by defective Numb or Neur partitioning during SOP division. These data raise the possibility that the defect originates later, perhaps in the course of cytokinesis.

### Notch is enriched at the apical pIIa-pIIb interface upon loss of AP-1 and Strat

**Fig. 3.**
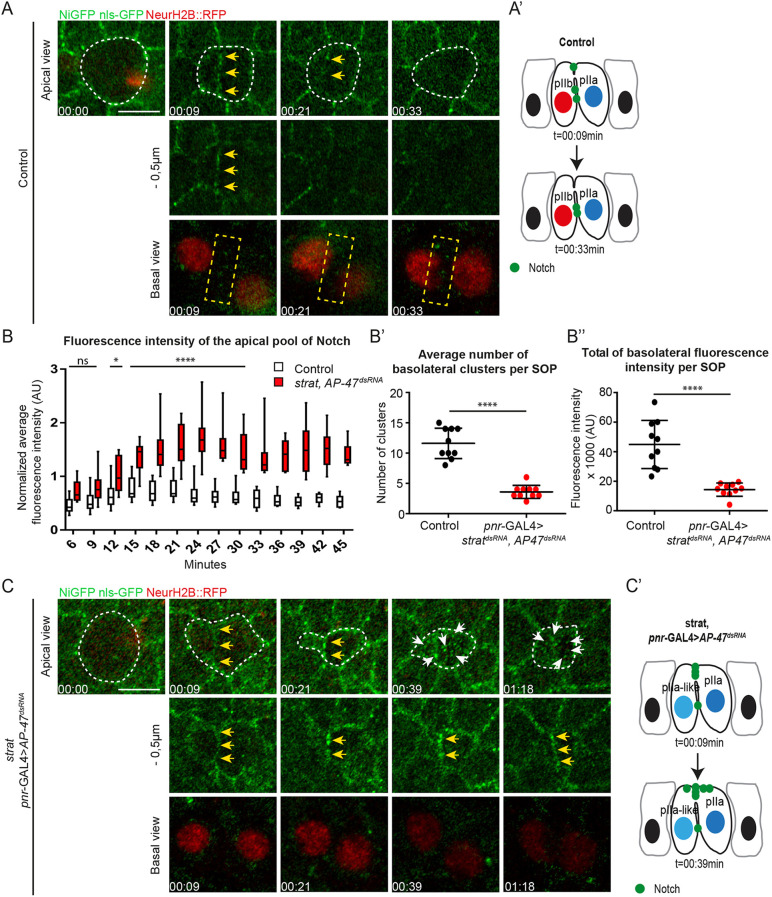
**Notch is enriched at the apical pIIa-pIIb interface in the absence of Strat and AP-1.** (A) Time-lapse imaging of NiGFP (green) and Histone2B::RFP expressed under the *neur* promoter (red) in dividing wild-type SOP (*n*=10). (A′) Schematic representation of Notch localization at t=9 min and t=33 min in wild-type SOP daughter cells. (B) Quantification of the fluorescence intensity of NiGFP at the apical pIIa-pIIb interface of wild-type SOP daughter cells (white) and *strat* SOP daughter cells expressing *pnr*-GAL4>*AP-47^dsRNA^* (red). Boxes extend from the 25th to 75th percentiles and the lines in the boxes represent the median. The whiskers span from the smallest value to the largest. The statistics were carried out from t=0 to 30 min and *n*=10 for both conditions (ns≥0.05; *P<0.05 and ****P<0.0001). Statistics were carried out until 30 min because most wild-type SOP movies stop at 30 min. (B′,B″) Quantification of the average number of NiGFP-Par3::scarlet-positive clusters (B′) and total fluorescence intensity of NiGFP (B″) at the lateral interface of wild-type SOP daughter cells (white, *n*=10) and SOP daughter cells expressing *pnr*-GAL4>*strat^dsRNA^, AP-47^dsRNA^* (red, *n*=10) between t21 and t35 min after the anaphase onset. Control (*n*=10) and *pnr*-GAL4>*strat^dsRNA^, AP-47^dsRNA^* (*n*=10) (see also Fig. S2). Data are mean±s.d. ****P<0.0001. (C) Time-lapse imaging of NiGFP (green) and Histone2B::RFP expressed under the *neur* promoter (red) in dividing *strat* SOPs expressing *pnr*-GAL4>*AP-47^dsRNA^* (*n*=10/12). (C′) Schematic representation of Notch localization at t=9 min and t=39 min in *strat* SOP daughter cells expressing *pnr*-GAL4>*AP-47^dsRNA^*. Dashed white lines highlight SOP and SOP daughter cells. Yellow arrows indicate the enrichment of NiGFP at the apical interface between SOP daughter cells and white arrows indicate apical compartments positive for NiGFP. Dashed yellow rectangles highlight compartments positive for NiGFP at the basolateral interface between wild-type SOP daughter cells. Basal views are a maximum projection of three confocal slices (Sum projection). Time is in h:min and the time 00:00 corresponds to the onset of anaphase in SOPs. Scale bar: 5 μm.

We next investigated the effect of AP-1 and Strat on the apico-basal distribution of Notch receptors. We monitored the dynamics of Notch bearing a GFP tag in its intracellular domain (Bellec et al., 2018, hereafter called NiGFP) throughout the asymmetric division of the SOP, identified by the nuclear marker Histone2B::RFP expressed under a minimal *neur* promoter (H2B::RFP, Fig. 3). Previously, elegant work from Schweisguth's laboratory identified two pools of Notch at the pIIa-pIIb interface: apical and basal to the midbody. The authors provide compelling evidence that the subset of receptors located basal to the midbody is the main contributor to signalling (Trylinski et al., 2017). In the control situation, we confirmed the presence of two pools of NiGFP along the apical-basal pIIa-pIIb interface (Fig. 3A,A′ and Fig. S2A). NiGFP is transiently detected at the apical pIIa-pIIb interface at ∼6-9 min after anaphase onset, with a signal intensity peaking 15-20 min before progressively disappearing at ∼30 min (Fig. 3A-B, Fig. S2A and Movie 1). Basal to the midbody, NiGFP localizes in lateral clusters, which appear ∼6-9 min following the anaphase onset and persist longer than the apical pool of NiGFP (up to 45 min after the anaphase onset; Fig. 3A-B″ and Fig. S2A,A′).

We found that NiGFP clusters colocalizes with Par3 at the apical (Fig. S2A, t=28 min) as well as at the lateral pIIa-pIIb interface (Fig. S2A), and our unpublished observations indicate that Par3 activity is required for the assembly of functional Notch signalling clusters during cytokinesis (Elise Houssin and R.L.B., unpublished). On average, 12 NiGFP-Par3 lateral clusters are detected in the wild type at t21 to t35 min (Fig. 3A,A′,B′ and Fig. S2A). Upon loss of Strat and AP-1, the number of NiGFP-Par3 lateral clusters and total intensity of NiGFP is decreased by 3.1-fold (Fig. 3B′,B″,C and Fig. S2B,B′). In parallel, we observed a 2.4-fold increase (mean intensity) in the amount of NiGFP at the apical pIIa-pIIb interface at t21 min after the onset of anaphase. This increase begins at ∼6-9 min after the onset of anaphase, and NiGFP signal persists for longer periods of time compared with the control (at least 78 min post-anaphase versus ∼30 min in the control; Fig. 3B-C′, Fig. S2B and Movie 2). In addition to accumulating at the apical interface, NiGFP also localizes in intracellular compartments present in the apical plane (white arrows, Fig. 3C,C′, and Movie 2). As the lateral clusters are strongly reduced upon loss of Strat and AP-1, our results lead us to hypothesize that the Notch gain-of-function phenotype could, at least in part, come from the apically enriched pool of NiGFP.

### Spdo, Neur and Delta are distributed with Notch at the apical pIIa-pIIb interface upon loss of Strat and AP-1

**Fig. 4.**
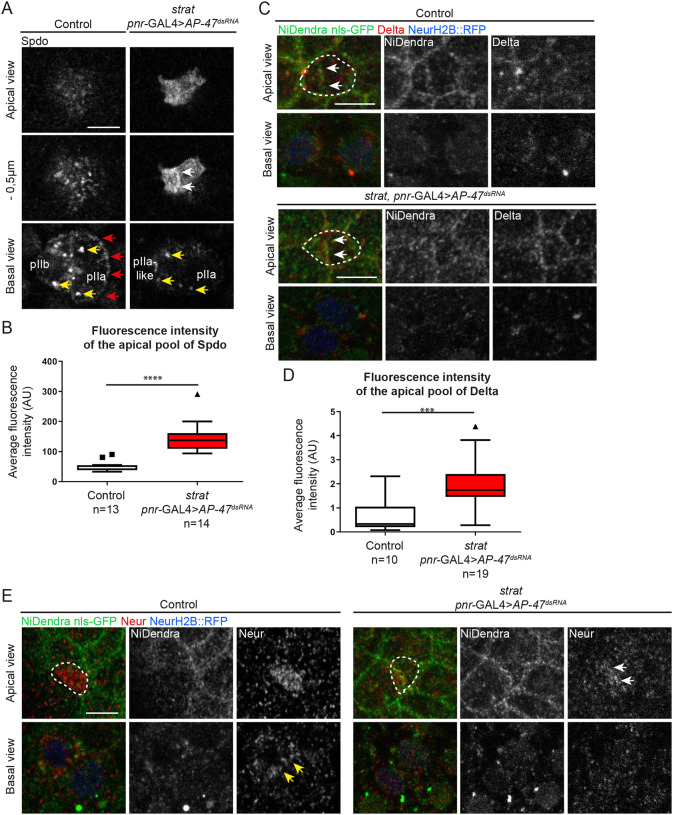
**Spdo, Neur and Delta are enriched at the apical pIIa-pIIb interface in the absence of Strat and AP-1.** (A) Localization of Spdo (anti-Spdo) in wild-type SOP daughter cells and in *strat* SOP daughter cells expressing *pnr*-GAL4>*AP-47^dsRNA^*. White arrows indicate the enrichment of Spdo at the apical interface between SOP daughter cells; red arrows indicate the enrichment of Spdo at the basolateral plasma membrane; yellow arrows indicate endosomes positive for Spdo. (B) Quantification of the fluorescence intensity of Spdo at the apical pole of wild-type and *strat* SOP daughter cells expressing *pnr*-GAL4>*AP-47^dsRNA^* (****P<0.0001). Boxes extend from the 25th to 75th percentiles, and the lines in the boxes represent the median. The squares and the triangle show maximum values. (C) Localization of NiDendra (anti-Dendra, green), Histone2B::RFP expressed under the *neur* promoter (blue) and Delta (anti-Delta, red) in wild-type SOP daughter cells and in *strat* SOP daughter cells expressing *pnr*-GAL4>*AP-47^dsRNA^*. White arrows indicate the enrichment of Notch and Delta at the apical interface between SOP daughter cells. A projection (maximum intensity) of the three most apical planes is shown for the apical view. (D) Quantification of the fluorescence intensity of Delta at the apical interface of wild-type and *strat* SOP daughter cells expressing *pnr*-GAL4>*AP-47^dsRNA^* (***P<0.001). Boxes extend from the 25th to 75th percentiles, and the lines in the boxes represent the median. The triangle shows the maximum value. (E) Localization of Neur (anti-Neur, green) and Histone2B::RFP expressed under the *neur* promoter (red) in wild-type SOP daughter cells (*n*=10) and in *strat* SOP daughter cells expressing *pnr*-GAL4>*AP-47^dsRNA^* (*n*=3). The same phenotype is observed in the *pnr*-GAL4>*strat^dsRNA^, AP-47^dsRNA^* (data not shown, *n*=9). Yellow arrows indicate the enrichment of Neur at the basolateral interface between SOP daughter cells; white arrows indicate the enrichment of Neur at the apical interface between SOP daughter cells. Dashed white lines highlight SOPs and SOP daughter cells. Scale bars: 5 μm.

Previously, *Notch* phenotypes observed in *AP-1* or *strat* mutant were associated with defects in Spdo and Delta localization. This prompted us to study the localization of Spdo and Delta on fixed specimens. As previously described, in the control situation, Spdo is faintly detected at the apical pole of SOP daughter cells and localizes predominantly in endosomes in the pIIb cell. In the pIIa cell, Spdo distributes not only in endosomes (yellow arrows, Fig. 4A) but also at the basolateral plasma membrane (red arrows, Fig. 4A; Cotton et al., 2013; Couturier et al., 2013; Hutterer and Knoblich, 2005; Langevin et al., 2005). In contrast, upon loss of Strat and AP-1, Spdo is still detected in dotted intracellular structures in SOP daughter cells but is no longer detected at the basolateral pIIa-pIIb interface (Fig. 4A). Instead, Spdo is enriched at the apical plasma membrane as well as at the apical pIIa-pIIb interface (*n*=12/14, white arrows, Fig. 4A,B).

Notch activation requires Neur-mediated Delta endocytosis. In the wild-type situation, Delta and Neur localize at the lateral pIIa-pIIb interface (Trylinski et al., 2017; Fig. 4C-E). Whereas Neur is uniformly distributed at the apical pole in cytokinesis (Trylinski et al., 2017; Fig. 4E), Delta is barely detected at the apical pIIa-pIIb interface (Fig. 4C,D). The loss of Strat and AP-1 does not appear to significantly alter the location of the Neur or Neur::GFP at the pIIa-pIIb interface (Figs 2D,D′ and 4E), but it results in an increase in the number of SOPs with Delta labelling at the apical pIIa-pIIb interface (60% of SOs against 44% in the control situation; Fig. 4C,D). Therefore, loss of Strat and AP-1 causes abnormally high levels of Notch/Delta and Spdo at the apical pIIa-pIIb interface at the expense of their localization to the basolateral interface. These observations raised the question of whether Notch activation can take place at the apical interface upon loss of Strat and AP-1. We tested the dynamics of the ectopic apical Notch pool using photobleaching. The apical pool of NiGFP in absence of Strat and AP-1 appears to be constantly replenished and rapidly recovered (yellow arrows, Fig. S3A). We noticed that 120 s after photobleaching, the fluorescence recovery reaches its maximum in control as well as upon loss of Strat and AP-1. However, the mobile fraction is around 67%±30% upon loss of Strat and AP-1 compared with 31%±15% in a control situation (Fig. S3B) with a t1/2 of around 40 s±7 s and a t1/2 of 37 s±11 s, respectively (Fig. S3C). The high recovery rate of the apical pool of NiGFP raises the question of its destiny and is in agreement with Notch activation that can take place at the apical interface upon loss of Strat and AP-1. To determine whether the *Notch* GOF observed in absence of Strat and AP-1 is ligand dependent, we inhibited Delta endocytosis using a stabilized version of Bearded that is acting as a strong antagonist of Neur (Brd^R^) (Perez-Mockus et al., 2017). Although SOs are composed of one neuron in a control situation, the overexpression of Brd^R^ resulted in a lateral inhibition defect and in a neurogenic phenotype, as previously reported (Bardin and Schweisguth, 2006; Lai et al., 2000; Fig. S3D). In absence of Strat and AP-1, the overexpression of Brd^R^ also induced a *Notch* loss-of-function phenotype: opposite to that observed upon loss of Strat and AP-1 only (Fig. S3D). These data demonstrate that the *Notch* gain-of-function phenotype observed in absence of Strat and AP-1 is ligand dependent. Together, our results suggest that, in the absence of Strat and AP-1, Delta-mediated Notch activation can take place at the apical interface of SOP daughter cells.

### The apical pool of Notch contributes to the *Notch* gain-of-function phenotype caused by the loss of Strat and AP-1

**Fig. 5.**
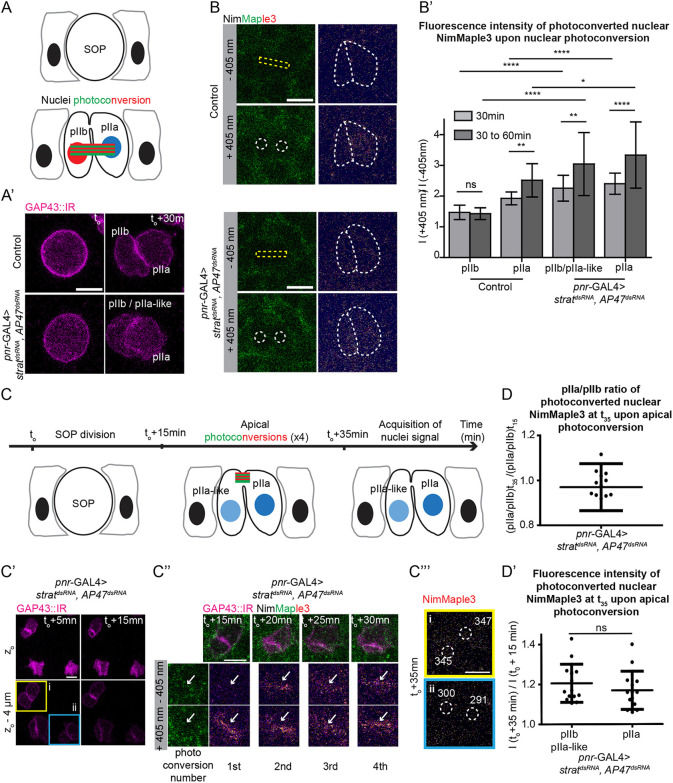
**Photoconverted apical Notch is detected in SOP daughter cell nuclei in the absence of Strat and AP-1.** (A,A′) Schematic representations and time-lapse imaging of wild-type and *pnr*-GAL4>*strat^dsRNA^, AP-47^dsRNA^* SOP daughter cells expressing GAP43::IR. Green and red rectangles represent the photoconversion area. (B) Photoconversion at t_o_+30 min of SOP daughter cells nuclei in wild-type and *pnr*-GAL4>*strat^dsRNA^, AP-47^dsRNA^*. Dashed yellow rectangles represent the photoconverted ROI; dashed white circles represent the area where nuclei signal has been measured; dashed white lines highlight SOP daughter cells. The red signal of NimMaple3 is measured before and after photoconversion. (B′) Histograms representing the fluorescence intensity of photoconverted nuclear signal in pIIb/pIIa-like and pIIa cells in wild-type and *pnr*-GAL4>*strat^dsRNA^, AP-47^dsRNA^* 30 min or between 30 and 60 min after anaphase onset (*n*=18 and *n*=14 for wild-type cells at 30 min and 30-60 min, respectively; *n*=24 and *n*=12 for *pnr*-GAL4>*strat^dsRNA^, AP-47^dsRNA^* cells at 30 min and 30-60 min). The *strat^dsRNA^, AP-47^dsRNA^* data were normalized according to Fig. S5A-B′,C-C‴. Photoconversion of apical NimMaple3 and nuclei signal measurement in *pnr*-GAL4>*strat^dsRNA^, AP-47^dsRNA^* SOP daughter cells. (C) Schematic representation of apical photoconversion assays in *pnr*-GAL4>*strat^dsRNA^, AP-47^dsRNA^* SOP daughter cells. Apical photoconversions were performed at 15, 20, 25 and 30 min after anaphase transition. Before photoconversion, *z*-stacks were generated to localize the apical interface using GAP43::IR. A *z*-stack was then acquired 35 min after the onset of anaphase to quantify nuclear NimMaple3. (C′) Time-lapse imaging of *pnr*-GAL4>*strat^dsRNA^, AP-47^dsRNA^* SOPs cells expressing GAP43::IR before apical photoconversions. Yellow square highlights the SOP where apical Notch is photoconverted; blue square highlights the SOP where Notch is not photoconverted and serves as reference. (C″) Photoconversion of apical NimMaple3 at t_o_ +15, 20, 25 and 30 min in *pnr*-GAL4>*strat^dsRNA^, AP-47^dsRNA^* SOP cells expressing GAP43::IR (*n*=14, four pupae). White arrows show Notch present at the apical pIIa-pIIb interface. (C‴) Measurements of nuclei signal (nuclei are delimited by dashed white circles) in photoconverted cells (upper panel, yellow) and non-photoconverted cells (lower panel, blue). pIIb or pIIa-like are localized at the left of the apical interface, and pIIa are localized at the right of the apical interface. (D) pIIa/pIIb ratio values of photoconverted nuclear NimMaple3 at t_o_+35 min upon apical NimMaple3 photoconversion in *strat^dsRNA^, AP-47^dsRNA^* (*n*=14, 4 pupae), with t_o_ corresponding to the onset of anaphase transition and t_15_ corresponding to the first photoconversion. (D′) Plot of normalized fluorescence intensity of the photoconverted nuclear signal in pIIb/pIIa-like and pIIa cells in *pnr*-GAL4>*strat^dsRNA^, AP-47^dsRNA^* (*n*=14, four pupae). Scale bars: 5 µm. t_o_ represents the SOP anaphase onset. In B′,D,D′, data are mean±s.d. (*P*≥0.05 is not significant, ns; **P<0.01). Because GAP43::IR is excluded from the nuclei, these were defined using an area within cells where the GAP43::IR signal background is the lowest.

To further investigate the ability of the apical pool of Notch to be cleaved and transferred into the nucleus, we photoconverted Notch receptors present at the membrane and measured the presence of photoconverted Notch in the nuclei of daughter cells using NimMaple3 (Trylinski et al., 2017). We first validated our ability to photoconvert nuclear NimMaple3 in the wild-type SOP daughter cells and in SOP daughter cells deprived of Strat and AP-1, 30 min after the anaphase onset (Fig. 5A,B). The plasma membrane of SOPs and daughter cells were identified using GAP43::IRFP670 (thereafter referred to as GAP43::IR) expressed under a minimal *neur* promoter (Fig. 5A′). As reported, in a control situation, higher levels of photoconverted nuclear Notch were found in the pIIa cell compared with the pIIb cell, thus reflecting the differential activation of the Notch signalling (Trylinski et al., 2017, Fig. 5B′). On the contrary, in absence of Strat and AP-1, the amount of photoconverted nuclear Notch is similar between the two daughter cells, at a level comparable with that of the nucleus of the control pIIa cell, in agreement with the *Notch* gain-of-function phenotype (Fig. 5B′). Once photoconverted, the nuclear signal rapidly decreased over time (t1/2=5 min), in agreement with the known instability of nuclear NICD (Fig. S5C,C′; Gupta-Rossi et al., 2001; Jarriault et al., 1995).

We then tested the precision of NimMaple3 photoconversion in controls. Photoconversion at the level of the apical pIIa-pIIb interface remained restricted to the apical plane as no undesired photoconversion occurred deeper in the cell (Fig. S4A-A″). In contrast, photoconversion within a ROI encompassing the lateral pIIa-pIIb plasma membrane interface led to unwanted photoconversion in the adjacent nuclei (Fig. S4B-B″) and at the apical level over a diameter of about 3-4 cells (Fig. S4C,C′). Therefore, the inability to ensure precise photoconversion on the lateral interface prevents us from using photoconversion to test whether Notch is activated from the apical, the lateral or from both pools of receptors in controls. However, this technical caveat is not a concern after loss of Strat and AP-1, as NimMaple3 and NiGFP signals are barely detected at the lateral interface (Fig. 3B′-C′ and Fig. S2B,B′). Following photonconversion of Notch at the apical pIIa-pIIb interface in this context, photoconverted NICD is present in the nuclei of both SOP daughter (Fig. 5C-C″). The levels of photoconverted Notch measured in both nuclei (Fig. 5C‴, cell *i* highlighted in yellow in Fig. 5C′) are higher than those found in the nuclei of a neighbouring SOP that divides at the same time, but was not exposed to photoconversion (Fig. 5C‴, cell *ii* highlighted in blue in Fig. 5C′). On average, the contribution of the apical pool of Notch when Strat and AP-1 are simultaneously impaired seems identical between the two nuclei, as demonstrated by the ratio of photoconverted nuclear signal pIIa/pIIa-like being close to 1 and the absence of significant differences in the amount of photoconverted nuclear signal between the two cells (Fig. 5D,D′). Taken together, these data argue that, in absence of Strat and AP-1, the apical enrichment of Notch at the interface provides a source of activated receptors present in nuclei of both daughter cells; this causes a *Notch* gain-of-function phenotype.

## DISCUSSION

### Strat and AP-1 act redundantly in the basolateral transport of Notch signalling components in SOPs

**Fig. 6.**
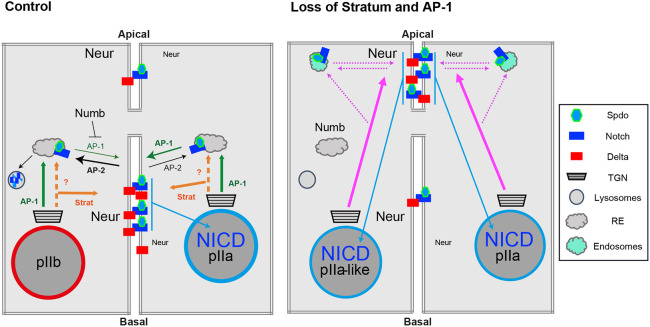
**Model for the roles of Strat and AP-1 in the transport of Notch signalling components.** Schematic representation of Strat and AP-1 function in the transport of Notch, Delta and Spdo in wild type and in absence of Strat and AP-1. Nuclei of cells are represented with a blue circle (Notch positive) or in red (Notch negative). AP-2, which is asymmetrically enriched in pIIb (Berdnik et al., 2002), promotes the endocytosis of Notch/Spdo (black arrows), while AP-1 regulates their recycling to basolateral membrane (green arrows), a step negatively regulated by Numb in pIIb cells. The trafficking of Notch, Delta and Spdo that is dependent on Strat or AP-1 is represented by orange or green arrows, respectively. In absence of Strat and AP-1, the mistrafficking of Notch, Delta and Spdo is represented by pink arrows. Notch and Spdo are also found in the apical dotted compartment, and their possible routing to and from there is depicted by a pink dashed arrow. TGN, *trans*-Golgi network; RE, recycling endosomes.

Previous work showed that Strat/Rab8 control the transport of Notch, Delta and Spdo from the TGN to the basolateral pole (Bellec et al., 2018), and that AP-1 controls the targeting of Notch and Spdo from the TGN to the basolateral membrane via the recycling endosomes (Benhra et al., 2011; Cotton et al., 2013). Here, we report that, upon concomitant loss of Strat and AP-1, Notch, Delta and Spdo are enriched at the apical pIIa-pIIb interface at the expense of lateral clusters (Fig. 6; Bellec et al., 2018; Benhra et al., 2011; Cotton et al., 2013). The defect of Notch and Delta localization observed upon loss of Strat and AP-47 is restricted to the SOP daughter cells, arguing that the SOP-specific genetic program somehow contributes to the trafficking defect. We found that AP-1 and Strat act redundantly to control the basolateral targeting of Notch, Delta and Spdo in SOP daughter cells, which explains the partial compensation of one by the other. These results are in agreement with previous studies demonstrating the involvement of Rab8, the activity and localization of which is controlled by Strat in *Drosophila* (Bellec et al., 2018; Devergne et al., 2017), in the basolateral sorting of proteins (Ang et al., 2003; Henry and Sheff, 2008; Huber et al., 1993). However, the role of Rab8 and AP-1 is not restricted to the basolateral transport, as the loss of one or the other has also been previously associated with defects in apical targeting and secretion in nematodes and vertebrates (Castillon et al., 2018; Gillard et al., 2015; Holloway et al., 2013; Nakajo et al., 2016; Norgate et al., 2006; Sato et al., 2014, 2007; Zhang et al., 2012). In our study, we described a thinner and less pigmented cuticle in the absence of Strat and AP-1. In contrast to the defect in Notch and Delta trafficking, the pigmentation defect is not restricted to the SOP. The cuticle phenotype could be caused by a defective AP-1 dependent transport of the Menkes Copper transporter ATP7a that regulates cuticle pigmentation (Holloway et al., 2013; Norgate et al., 2006) and/or by a reduction in apical secretion of cuticle components that may rely on AP-1 function as does the apical glue granule secretion in salivary glands (Burgess et al., 2011). These apparent contradictory results might be explained by a distinct Rab8- or AP-1-mediated sorting, depending on the cargo to be transported or on the tissue. In addition, sorting might occur at the level of RE. If a protein that does not normally transit via RE is found to be present in RE in a mutant background, it may be sorted and transported to an unusual apical location by default.

### Contribution of the apical pool to Notch activation upon loss of AP-1 and Strat

Upon loss of Strat and AP-47, Notch, Spdo, Delta are present in higher amounts at the apical pIIa-pIIb interface where Neur is also present. Our photo-tracking of photoconverted NimMaple3 indicates that Notch activation takes place bi-directionally from the apical pIIa-pIIb interface upon loss of Strat and AP-47. Although it is possible that the residual lateral clusters also contribute to Notch activation upon loss of AP-1 and Strat, our data argue that the apical pool becomes the main contributor. Whether Notch activation can also take place from the Notch-positive compartments observed at the apical pole of SOP daughter cells upon loss of Strat and AP-1 remains an unresolved issue. Likewise, we cannot firmly rule out that, upon loss of Strat and AP-47, the apical pool of NiGFP is relocated to the lateral pIIa-pIIb interface where Notch proteolytic activation has been reported to take place (Couturier et al., 2012; Trylinksi et al., 2017). In any case, the formal proof needed to determine the subcellular site of Notch activation awaits the development of methods to allow the direct monitoring of *in vivo* S2 cleavage.

However, the fact that Sec15, a subunit of the exocyst complex, and Arp2/3-WASp (Actin-related protein 2/3-Wiskott-Aldrich syndrome protein) are involved in apical trafficking of Spdo and Delta, respectively, and are required for Notch-Delta signalling is consistent with the proposal of apical activation of Notch (Jafar-Nejad et al., 2005; Rajan et al., 2009). Neur has been reported to trigger basal-to-apical transcytosis of Delta, although the physiological consequence in terms of Notch signalling was not demonstrated (Benhra et al., 2010). Altogether, these studies indicate that Notch activation can occur from both the basal and the apical pIIa-pIIb interface. However, the relative contribution of the two pools might be context dependent. Indeed, the minor contribution of the apical pool of receptors to the Notch activation described in the control situation (Trylinski et al., 2017) suggests that apical activation may become preponderant when a given threshold of Notch, Delta and Spdo is reached, as described here in *strat* SOs depleted of AP-1. Although Notch activation can take place basally as well as apically (Trylinski et al., 2017; this study), the relative efficiency, strength and duration of lateral versus apical signalling is currently unknown. Another parameter to consider is the distance between the site of proteolytic activation and the nucleus. Compared with the apical interface, the lateral interface is located at a short distance from the nucleus, potentially reducing the time lag between NICD production and the transcriptional response.

### Loss of Strat and AP-1 causes Notch activation in both SOP daughter cells

Loss of Strat and AP-1 leads to a *Notch* gain of function despite the unequal partitioning of Numb. This phenotype can be explained by the lack of AP-1 that prevents Numb from repressing the Notch-Spdo recycling normally driving endosomal degradation of Notch-Spdo (Cotton et al., 2013; Couturier et al., 2013; Johnson et al., 2016). Thus, Numb is not able to repress Notch activation upon loss of AP-1 and Strat, and is therefore unable to trigger symmetry breaking in Notch signalling at SOP cytokinesis.

Proteolytic activation of Notch requires the binding of the ligand and its subsequent Neur-mediated endocytosis. Although Neur is unequally partitioned during SOP division both in control and upon loss of Strat, Neur also localizes at the apical cortex of both SOP daughter cells and at the pIIa-pIIb interface following SOP division. As Delta and Neur localize at the apical interface between SOP daughter cells, and Notch signalling is ligand dependent upon loss of Strat and AP-1, we propose the hypothesis that Neur can promote endocytosis of Delta in both daughter cells, thus leading to bi-directional Notch proteolytic activation. In agreement with this proposal, according to which Neur is active in the two SOP daughter cells, it is interesting to note that, in *numb* mutant cells, despite the unequal inheritance of Neur, Delta-dependent activation of Notch takes place in both daughter cells (Le Borgne and Schweisguth, 2003), as observed here upon loss of Strat and AP-1.

In conclusion and in line with previous reports (Trylinski et al., 2017), our study further illustrates how the apico-basal trafficking of Notch, Spdo and Delta along the pIIa-pIIb interface contributes to private communication between two daughter cells embedded in a monolayer epithelium at cytokinesis. The issue of whether the mechanism is conserved awaits further investigation, but given the key role played by Notch in the acquisition of cell fate in vertebrates, it is conceivable that the regulation described in this study may be general.

## MATERIALS AND METHODS

### *Drosophila* stocks and genetics

*Drosophila melanogaster* stocks were maintained and crossed at 25°C. Mitotic clones were induced using the FLP-FRT technique using the *hs*-FLP and by heat shocking (2×60 min at 37°C) at second and early third instar larvae stages. *pnr*-GAL4 was used to drive the expression of the *AP-47^dsRNA^* and the *strat^dsRNA^*. The following stocks were used in this study.

Fig. 1B: *y, hs*-FLP (/*w*); *Ubi-GFP nls,* FRT40A/*Ubi-GFP nls*, FRT40A and *y, w, hs*-FLP (/*w^−^*); *Ubi-GFP nls,* FRT40A/*strat,* FRT40A; *pnr-*GAL4/*AP-47^dsRNA^* (stock #24017 from Vienna Drosophila Resource Center, *w^1118^*; P(GD14206)v24017/TM3)

Table 1: *y, hs*-FLP (/*w*); *Ubi-GFP nls,* FRT40A/*Ubi-GFP nls*, FRT40A and *y, hs*-FLP (/*w*); *Ubi-GFP nls,* FRT40A/*strat,* FRT40A and *y, w, hs*-FLP (/*w*); *Ubi-GFP nls,* FRT40A/*strat,* FRT40A; *pnr-*GAL4/*AP-47^dsRNA^* and *y, w, NimMaple3* (a kind gift from F. Schweisguth, Institut Pasteur, Paris, France Trylinski et al., 2017)/Y; *strat^dsRNA^*/+; *pnr-*GAL4, *neur*-GAP43::IRFP670/*AP-47^dsRNA^*

Fig. 2A: *Cad::GFP*/+; *pnr-*GAL4/+; +/CyO::CFP; *pnr*-GAL4/+ and *y, w, hs*-FLP (/*w*); *Ubi-GFP nls,* FRT40A/*strat,* FRT40A; *pnr-*GAL4/*AP-47^dsRNA^*

Fig. 2B: *y*, *hs*-FLP (*w*); *Ubi-RFP nls*, FRT40A/*Numb*::*GFP*^crispr^, FRT40A

Fig. 2B′: w, hs-FLP *neur-H2B*::RFP/*w*; *Ubi-GFP nls,* FRT40A/*strat, Numb*::*GFP*^crispr^, FRT40A; *pnr-*GAL4/*AP-47^dsRNA^*

Fig. 2C: *y, w, hs*-FLP/*NiDendra*, *neur-H2B*::RFP; *Ubi-GFP nls,* FRT40A/FRT40A; *pnr-*GAL4/+

Fig. 2C′: y, w, hs-FLP/*NiDendra*, *neur-H2B*::RFP; *Ubi-GFP nls,* FRT40A/*strat,* FRT40A; *pnr-*GAL4/*AP-47^dsRNA^*

Fig. 2D: y, w, PB[y+ attP-3B Neur::GFP] 22A3] *neur*-iRFP670nls (a kind gift from F. Schweisguth; Couturier et al., 2014; Perez-Mockus et al., 2017)/+; P [attP2 *neur*-GAP43-iRFP 68A4] (this study)*, pnr-*GAL4/*+*

Fig. 2D′: y, w, PB[y+ attP-3B Neur::GFP] 22A3] *neur*-iRFP670nls/*strat^dsRNA^*; P [attP2 *neur*-GAP43-iRFP 68A4]*, pnr-*GAL4/*AP-47^dsRNA^*

Fig. 3: y, w, hs-FLP/*NiGFP*, *neur-H2B*::RFP; *Ubi-GFP nls,* FRT40A/FRT40A; *pnr-*GAL4/+ and *y, w, hs*-FLP/*NiGFP*, *neur-H2B*::RFP; *Ubi-GFP nls,* FRT40A/*strat,* FRT40A; *pnr-*GAL4/*AP-47^dsRNA^* and *y, w, NiGFP*, *Par3::Scarlet*; *+/+*; *pnr-*GAL4/*+* and *y, w, NiGFP*, *Par3::Scarlet*;*strat^dsRNA^/+*; *pnr-*GAL4/*AP-47^dsRNA^*

Fig. 4A,B: W1118 and *y, w, hs*-FLP (/*w*); *Ubi-GFP nls,* FRT40A/*strat,* FRT40A; *pnr-*GAL4/*AP-47^dsRNA^*

Fig. 4C,D: y, w, hs-FLP/*NiDendra*, *neur-H2B*::RFP; *Ubi-GFP nls,* FRT40A/FRT40A; *pnr-*GAL4/+ and *y, w, hs*-FLP/*NiDendra*, *neur-H2B*::RFP; *Ubi-GFP nls,* FRT40A/*strat,* FRT40A; *pnr-*GAL4/*AP-47^dsRNA^*

Fig. 4E: y, w, hs-FLP/*NiDendra*, *neur-H2B*::RFP; *Ubi-GFP nls,* FRT40A/FRT40A; *pnr-*GAL4/+

*y, w, hs*-FLP/*NiDendra*, *neur-H2B*::RFP; *Ubi-GFP nls,* FRT40A/*strat,* FRT40A; *pnr-*GAL4/*AP-47^dsRNA^*

and *y, w, NiGFP*, *Par3::Scarlet*; *strat^dsRNA^*/+; *pnr-*GAL4/*AP-47^dsRNA^*

Fig. 5: *y, w, NimMaple3*/Y; *strat^dsRNA^*/+; *pnr-*GAL4, *neur*-GAP43::IRFP670/*AP-47^dsRNA^*

Fig. S1A,A′: *NiDendra*, *neur-H2B*::RFP; +/+

Fig. S1B,B′: y, w, hs-FLP/*NiDendra*, *neur-H2B*::RFP; *Ubi-GFP nls,* FRT40A/*strat,* FRT40A; *pnr-*GAL4/*AP-47^dsRNA^*

Fig. S1C: y, w, hs-FLP (/*w^−^*); *Ubi-GFP nls,* FRT40A/*strat,* FRT40A; *pnr-*GAL4/*AP-47^dsRNA^*

Fig. S1D: y, w, NiGFP, *Par3::Scarlet* (a kind gift from J. Januschke, University of Dundee, UK); *+/+*; *pnr-*GAL4/*+* and *y, w, NiGFP*, *Par3::Scarlet*; *strat^dsRNA^*/+; *pnr-*GAL4/*AP-47^dsRNA^*

Fig. S2: y, w, NiGFP, *Par3::Scarlet*; *+/+*; *pnr-*GAL4/*+* and *y, w, NiGFP*, *Par3::Scarlet*;*strat^dsRNA^/+*; *pnr-*GAL4/*AP-47^dsRNA^*

Fig. S3A: *y, w, NiGFP*, *Par3::Scarlet*; *strat^dsRNA^*/+; *pnr-*GAL4/*AP-47^dsRNA^*

Fig. S3B,C: *y, w, N^iGFP−5^, neur-H2B*::RFP (control; a kind gift from F. Schweisguth; Couturier et al., 2012) and *y, w, NiGFP*, *Par3::Scarlet*; *strat^dsRNA^*/+; *pnr-*GAL4/*AP-47^dsRNA^*

Fig. S3D: *y,w, NiGFP*; *+/+*; *pnr-*GAL4/*+*

*y, w, NiGFP*; *strat^dsRNA^/+*; *pnr-*GAL4/*AP-47^dsRNA^*

*y, w*; +/+; *UAS-Brd^R^* (a kind gift from F. Schweisguth; Perez-Mockus et al., 2017)*, pnr-*GAL4/*+*

and *y, w*; *strat^dsRNA^/+*; *UAS-Brd^R^, pnr-*GAL4/*AP-47^dsRNA^*

Fig. S4A-C′: *y, w, NimMaple3*/Y; +/+ *pnr-*GAL4, *neur*-GAP43::IRFP670/+

Fig. S5: *y, w, NimMaple3*/Y; +/+ *pnr-*GAL4, *neur*-GAP43::IRFP670/+ and *y, w, NimMaple3*/Y; *strat^dsRNA^*/+; *pnr-*GAL4, *neur*-GAP43::IRFP670/*AP-47^dsRNA^*

Movie 1: *y, w, hs*-FLP/*NiGFP*, *neur-H2B*::RFP; *Ubi-GFP nls,* FRT40A/FRT40A; *pnr-*GAL4/+

Movie 2: y, w, hs-FLP/*NiGFP*, *neur-H2B*::RFP; *Ubi-GFP nls,* FRT40A/*strat,* FRT40A; *pnr-*GAL4/*AP-47^dsRNA^*

### Immunofluorescence and antibodies

Pupae were aged for 16.5 to 18.5 h after puparium formation (APF) for SOPs and SOPs daughter cell analysis, and were aged for 24 h to 28 h APF for lineage analysis. Pupae were dissected in 1× phosphate-buffered saline (1×PBS) and then fixed for 15 min in 4% paraformaldehyde at room temperature. Dissection and staining conditions were essentially as previously described (Le Borgne and Schweisguth, 2003). Primary antibodies used were rat anti-Elav [7E10, Developmental Studies Hybridoma Bank (DSHB), 1:200], goat anti-Su(H) (sc15813, Santa Cruz, 1:500), mouse anti-Cut (2B10, DSHB, 1:500), rabbit anti-Neur (Lai et al., 2001), goat anti-Numb (SC23579, Santa Cruz, 1:200), rabbit anti-Spdo (a kind gift from J. Skeath, Washington University School of Medicine, St Louis, MO, USA; 1:2000) (O'Connor-Giles and Skeath, 2003), mouse anti-DECD (C594.9B, DSHB, 1:200), mouse anti-Cora (C615.16, DSHB, 1:500), rat anti-DE-Cad (DCAD2, DSHB, 1:500) and rabbit anti-Dendra (antibodies-online.com, ABIN361314, 1:1000). Cy2-, Cy3- and Cy5-coupled secondary antibodies (1:400) were from Jackson Laboratories (donkey anti-goat, 705-225-147, 705-165-147 and 705-175-147; donkey anti-mouse min cross react with rat, 715-225-151, 715-165-151 and 715-175-151; goat anti-rabbit, 111-225-144, 111-165-144 and 111-175-144; donkey anti-rat min cross react with mouse, 712-225-153, 712-165-153 and 712-175-153).

### Generation of NiDendra and *neur*-GAP43::iRFP670

The NiDendra construct was generated using the CRISPR/Cas9 method as previously described (Gratz et al., 2013a,b). As for the NiGFP construct, the following gRNAs were used: 5′-AACTTGAATGGATTGAACCCGGG-3′ and 5′-CGAACTGGAGGGTTCTCCTGTTG-3′ to introduce Cas9 cuts in exon 6 (Bellec et al., 2018). The Dendra and the 3xP3-DsRed cassette flanked by GVG linkers and by loxP, respectively, were introduced at the previously described position (NiYFP4) (Couturier et al., 2012). Homology arms 1 and 2 were of 1064 bp and 1263 bp in length, respectively. Injection was performed by Bestgene in the *yw*; attP40(nos-cas9)/Cyo stock. The correct position of the Dendra and the DsRed cassette was verified by PCR and sequencing. The DsRed was then removed by crossings with *if*/Cyo, Cre, *w* stock. The insertion of the Dendra tag did not alter the functionality of Notch. Anti-Dendra signal is detected in the pIIa nucleus, indicating that processed NiDendra is translocated into the nucleus (data not shown).

The *neur*-GAP43::iRFP670 transgene was generated by fusing the iRFP670 gene (Shcherbakova and Verkhusha, 2013) to the GAP43 palmitoylation sequence (Mavrakis et al., 2009). The resulting gene was inserted into the Stinger-attB-p*neur*-GFP plasmid (Aerts et al., 2010) to replace GFP. After sequencing, the resulting transgene was integrated at the P (CaryP)attP2 at position 68A. Transgenesis was performed by Bestgene.

### Imaging

Images of fixed nota were acquired with a Leica SPE confocal microscope and a Zeiss Airyscan microscope (LSM 880 with AiryScan module). Live imaging of NiGFP with Par3::Scarlet was performed with a Leica SPE confocal microscope. Live imaging of NiGFP in wild-type SOP and in *strat* SOP expressing *pnr*-GAL4>*AP-47^dsRNA^* (Fig. S2) was performed with a Zeiss Airyscan microscope (LSM 880 with AiryScan module). All images were processed and assembled using ImageJ 1.48 and Adobe Illustrator.

### Quantification of the enrichment of Notch at the apical and basal interface

To quantify the signal of NiGFP at the apical interface, we measured the signal in a manually drawn area on sum slices of the two apical planes where the Notch signal at the apical interface is the strongest. We also measured the signal between two epithelial cells within an equivalent drawn area and on the same sum slices. We then calculated the following ratio: average fluorescence intensity at the apical interface between SOP daughter cells/average fluorescence intensity at the apical interface between epithelial cells. This ratio was calculated for each time point. In absence of AP-1 and Strat, the manually drawn area was minimized to avoid considering the apical punctate structures positive for Notch.

To quantify the signal of NiGFP at the basal interface of SOP daughter cells, we first manually identified each individual NiGFP and Par3::Scarlet-positive cluster present in every confocal section (thickness 0.5 μm) as shown in Fig. S2A′,B′. For each movie, we determined the time at which the largest number of NiGFP-Par3 clusters was counted (between t21 and t35 min). When a cluster was detected on two consecutive optical sections, it was considered as one cluster. To determine the intensity of NiGFP per cluster, a line two pixels in width and 20 pixels long encompassing the middle of the cluster was drawn, as shown in Fig. S2A″,B″. The corresponding plot profile was obtained on Image J and allowed us to determine the fluorescence intensity within the cluster and the fluorescence intensity of the background (corresponding to the average of the minimal values of the 20 pixel long line on both sides of the fluorescence peak corresponding to the cluster). After removing the background value, the integration of the signal under the curve gave us the value of the NiGFP signal present in the cluster. For each organ, we have determined the number of clusters and their intensity. By adding the intensity of each cluster, we obtained the total NiGFP intensity at the lateral pIIa-pIIb interface.

### Quantification of the apical enrichment of Spdo and Delta

The fluorescence intensity was calculated with ImageJ, at the two-cell stage, as previously described (Bellec et al., 2018). For Spdo, the average fluorescence intensity was measured in a manually drawn area on sum slices of the two most-apical planes, where Spdo is enriched. The background noise was measured in the same way and was subtracted from the apical intensity value. For the Delta, the same protocol was applied: the average fluorescence intensity was measured in a manually drawn area on sum slices of the two apical planes where Delta is enriched at the interface between the two daughter cells. These values were divided by the average intensity measured at the apical interface between epidermal cells, on the same sum slices and with the same manually drawn area. The background noise was measured in the same way and was subtracted from the apical intensity values.

### FRAP experiments

Photobleaching of NiGFP/NiGFP5 was performed using a Zeiss AiryScan (LSM 880 with AiryScan module) with the 488 nm laser wavelength (20 mW) at 90% of the maximal power and by using the 63× oil objective (NA 1.4). Two consecutive iterations were performed. The FRAP area was defined by the pIIa/pIIb interface. A control area corresponding to an adjacent epidermal cell interface was measured to obtain the general photobleaching of the sample over the period of acquisition. All FRAP data were analysed using the easyFRAP software tool (https://easyfrap.vmnet.upatras.gr/?AspxAutoDetectCookieSupport=1). Half-time (t1/2) and mobile fraction were then extracted with GraphPAD Prism software using a one-component equation.

### Photoconversion experiments

Photoconversion of NimMaple3 was performed using a Zeiss AiryScan (LSM 880 with AiryScan module) with a 405 nm diode (30 mW) at 1.8% power (40 iterations and pixel dwell time: 1.52 µs) according to Trylinski et al. (2017). In nuclear photoconversion assays, the photoconverted ROI (Fig. 5B, dashed yellow rectangles) were defined using the GAP43::IR expressed under the *neur* promoter. Because GAP43::IR is excluded from the nuclei, nuclei were defined using an area within cells where the GAP43::IR signal background is the lowest. Nuclei measurements (dashed white circles, Fig. 5B,C‴) were then performed using a circular ROI corresponding to the half of the presumptive diameter of nuclei with the centre of the ROI positioned on the centre of mass of nuclei.

Apical photoconversions in *pnr*-GAL4>*strat^dsRNA^, AP-47^dsRNA^* SOP daughter cells were performed at 15, 20, 25 and 30 min after anaphase transition. Before photoconversion, *z*-stacks were performed to localize the apical interface using GAP43::IR only first, and then with green NimMaple3. A *z*-stack was then acquired 35 min after the anaphase onset to quantify nuclear NimMaple3 in photoconverted SOP (Fig. 5C′,C″i) and adjacent non photoconverted SOP (Fig. 5C′,C″ii). No threshold has been applied. The measurements of the photoconverted nuclear NimMaple3 intensity upon apical photoconversion (Fig. 5D′) at t35 were performed by normalizing the fluorescence ratio I (to+35 min)/I (to+15 mn) of the photoconverted SOP by the fluorescence ratio I (to+35 min)/I (to+15 mn) of the non-photoconverted SOP.

The NimMaple3 signal at the apical interfaces between epidermal cells in the wild-type situation is, on average, 1.59-fold higher than upon silencing of *strat* and *AP-47* (Fig. S5A,A′), and leads to higher photoconversion efficiency (Fig. S5B,B′). A normalization factor (1.59) was applied on nuclei photoconversion assays (Fig. 5B′, *strat^dsRNA^*, *AP47^dsRNA^* condition).

### Statistical analysis

Statistical analyses were carried out using the GraphPad Prism 6.07 software. A two-way ANOVA was performed for the quantification of the enrichment of Notch at the apical and at the basal pIIa-pIIb interface with a multiple comparison Bonferroni test. For other quantifications, we performed a *t*-test, if the data followed a normal distribution, or an unpaired Wilcoxon test or an unpaired Mann–Whitney test, if the data did not follow a normal distribution. The normal distribution was tested by a Shapiro test. Statistical significances are represented as follows: not significant (ns)≥0.05; *P<0.05; **P<0.01; ***P<0.001 and ****P<0.0001.

## Supplementary Material

Supplementary information
