# Supplementary material for: The Clathrin adaptor AP-1 and Stratum act in parallel pathways to control Notch activation in Drosophila sensory organ precursors cells
Source: Development. 2021 Jan 11;148(1):dev191437. doi: 10.1242/dev.191437 (PMC7823167; doi:10.1242/dev.191437)
Supplement: Supplementary information [file develop-148-191437-s1.pdf]

Supplementary Material

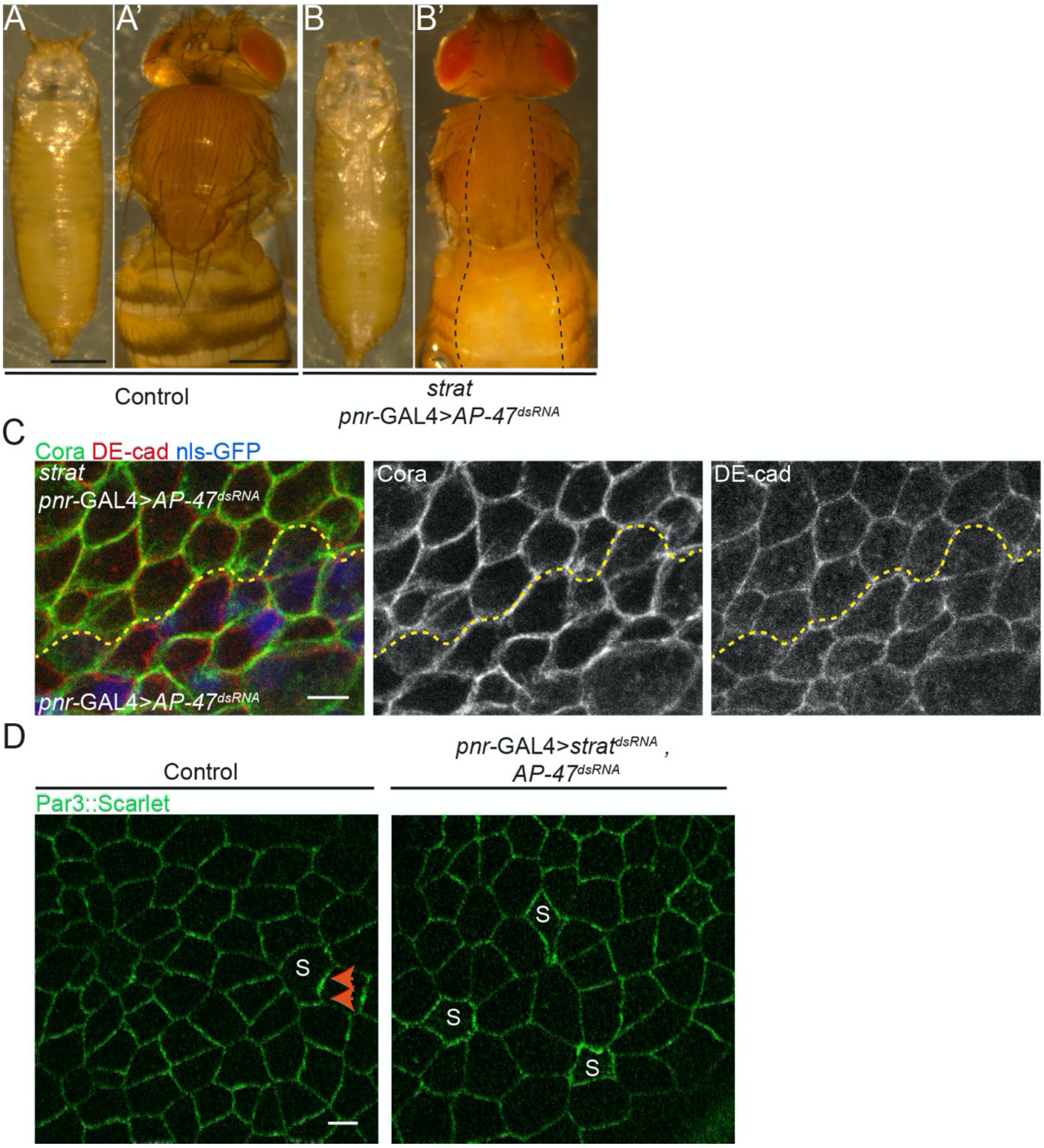

Figure S1: Apical basal polarity is maintained upon loss of Strat and AP-1

A-B'. Pictures of NiDendra (**A-A'**) and NiDendra, *strat* expressing *pnr*-GAL4>*AP-47*^{dsRNA} (**B-B'**) individuals at the pupal and adult stages. Dashed black lines highlight depigmented areas. **C.** Localization of Coracle (anti-Cora, green) and DE-Cadherin (anti-DE-cad, red) in wild-type clone expressing *pnr*-GAL4>*AP-47*^{dsRNA} and in *strat* clone expressing *pnr*-GAL4>*AP-47*^{dsRNA}. Dashed yellow lines delineate *strat* clones. **D.** Localization of Par3 (Par3::Scarlet, green) in wild-type and in *pnr*-GAL4>*strat*^{dsRNA}, *AP-47*^{dsRNA}. N=14 wild-type SOPs and n=16 SOPs expressing *pnr*-GAL4>*strat*^{dsRNA}, *AP-47*^{dsRNA}. Orange arrows point to the enrichment of Par3 at the posterior pole. S represents SOPs. Scale bars are 500 µm for pictures and 5 µm for immunostainings.

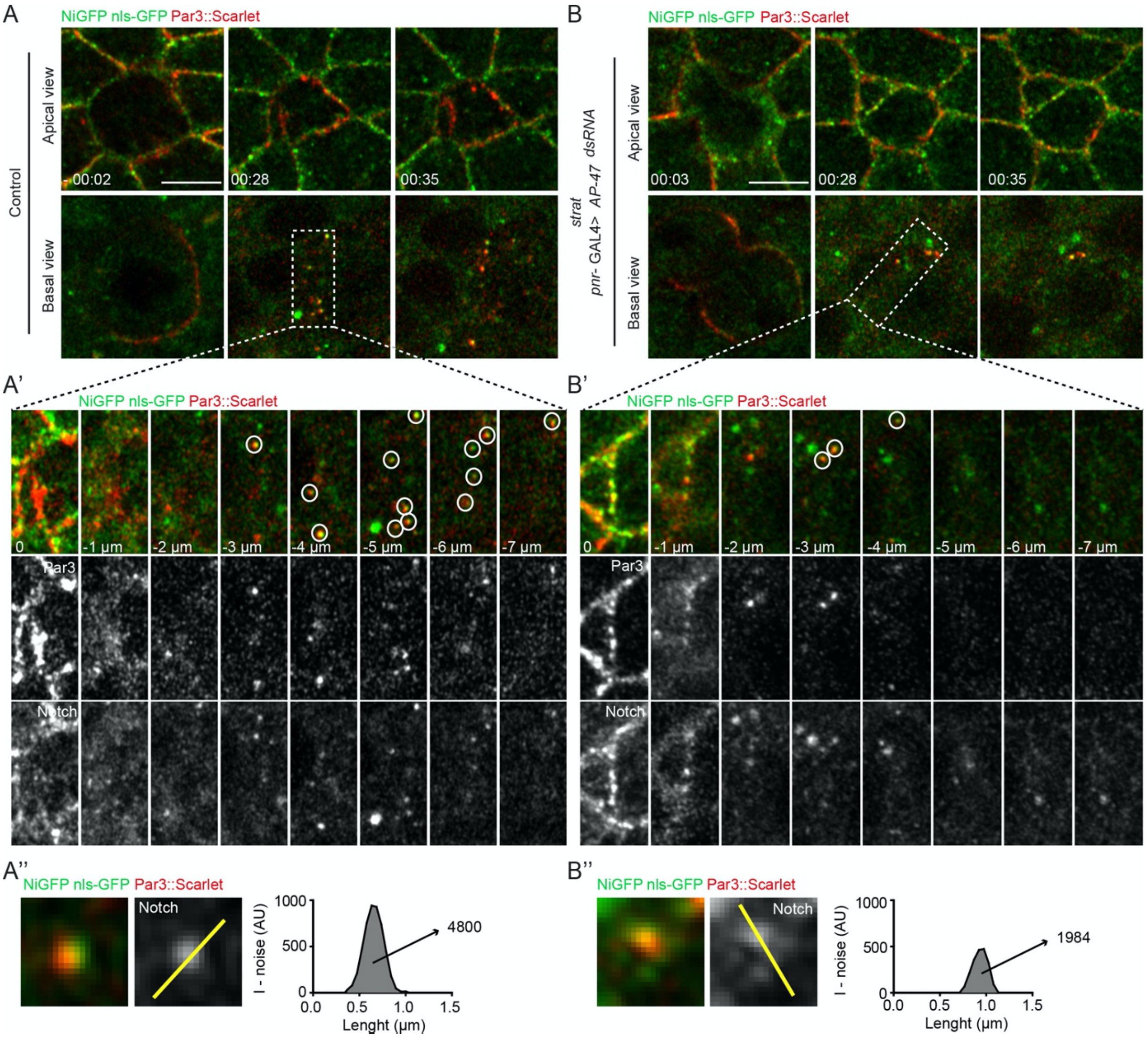

Figure S2: Notch is enriched at the apical pIIa-pIIb interface in the absence of Strat and AP-1

A-B. Time-lapse imaging of NiGFP (green) and Par3::Scarlet (red) in dividing wild-type (**A**, n=11) or *strat* SOP expressing *pnr*-GAL4>*strat*^{dsRNA}, *AP-47*^{dsRNA} (**B**, n=22). Dashed white rectangles highlight compartments positive for NiGFP and Par3::Scarlet at the basolateral interface between wild-type SOP daughter cells. **A'-B'.** Distribution of the NiGFP and Par3::Scarlet-positive clusters detected at the pIIa-pIIb interface at t28min of time-lapse presented in **A** and **B**, respectively. For the quantitation of the intensity of the lateral NiGFP, only the NiGFP-Par3::Scarlet positive clusters located between -3µm and -7µm (surrounded by a white circle and basal relative to the mibody, whose position was determined separately using Sqh::RFP as a marker as reported in (Daniel et al, 2018)) were considered, with 0µm corresponding to the level of adherens junctions. **A", B".** Quantitation of the Notch signal presents within one cluster. The yellow lines delineate the position of the plot profile. Time is in hour:minute and the time 00:00 corresponds to the SOP anaphase onset. Scale bar is 5 µm.

Development • Supplementary information

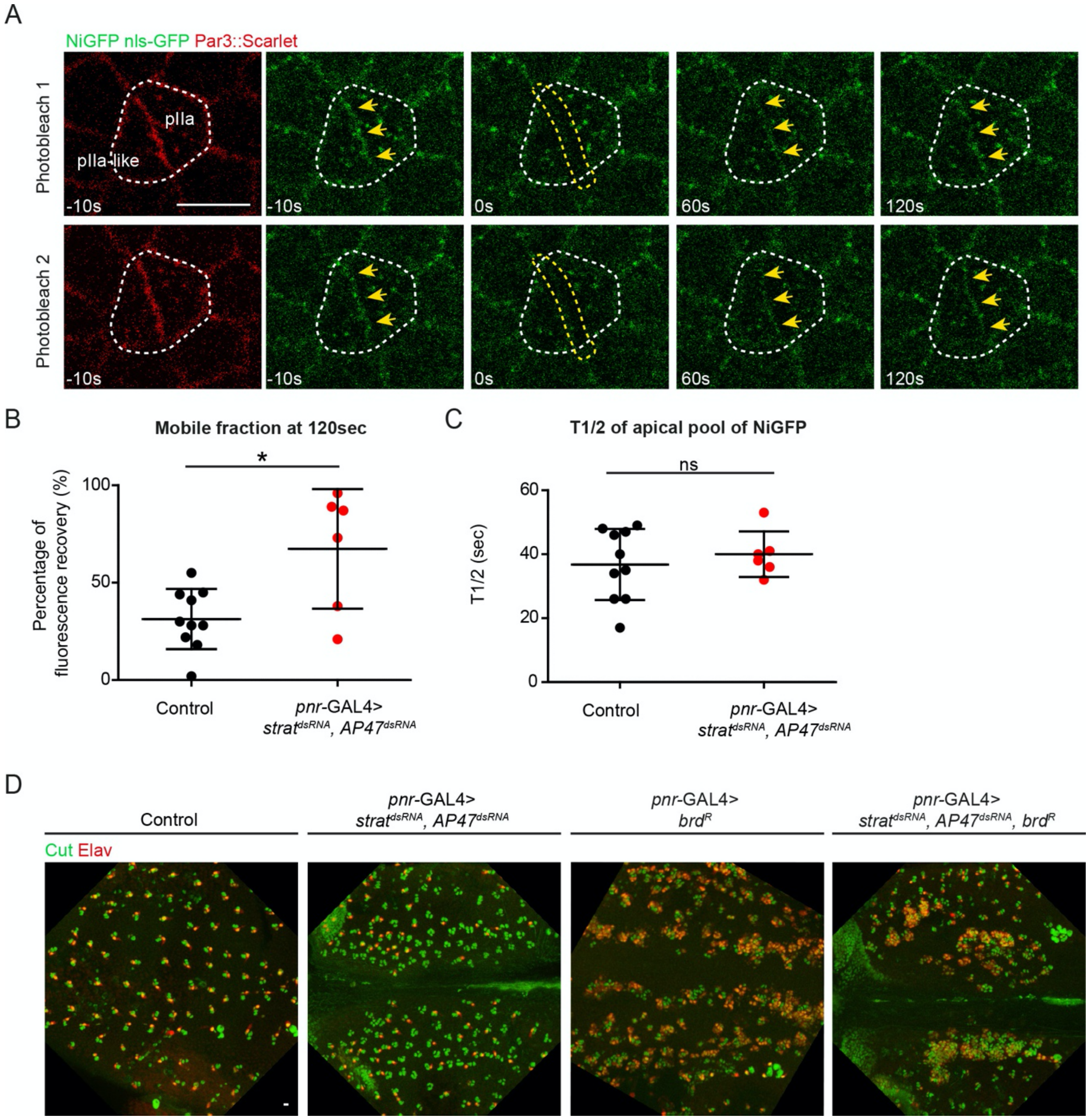

Figure S3: The apical pool of Notch is highly dynamic

A. Time-lapse imaging of NiGFP (green) and Par3::Scarlet (red) after two consecutive cycles of photobleaching of Notch at the apical pIIa-pIIb interface in *pnr*-GAL4>*strat*^{dsRNA}, *AP-47*^{dsRNA} conditions. Time is in seconds. Dashed white lines highlight the SOP daughter cells and yellow arrows point to the enrichment of NiGFP at the apical interface between SOP daughter cells. Dashed yellow lines delineate the photobleached area. **B.** Plot of the mobile fraction observed at 120 seconds post photobleaching at the apical interface of wild-type and *pnr*-GAL4>*strat*^{dsRNA}, *AP-47*^{dsRNA} SOP daughter cells (**P*<0.05). **C.** Plot of t1/2 of fluorescence recovery of the apical pool of NiGFP in wild-type and *pnr*-GAL4>*strat*^{dsRNA}, *AP-47*^{dsRNA} SOP daughter cells (ns≥0.05). **D.** Maximal projection of confocal sections of a pupal notum at 28h APF of the indicated genotype. Cells composing the SO were identified with Cut (anti-Cut, green) and neurons were identified with Elav (anti-Elav, red). Scale bar is 5 µm.

Development • Supplementary information

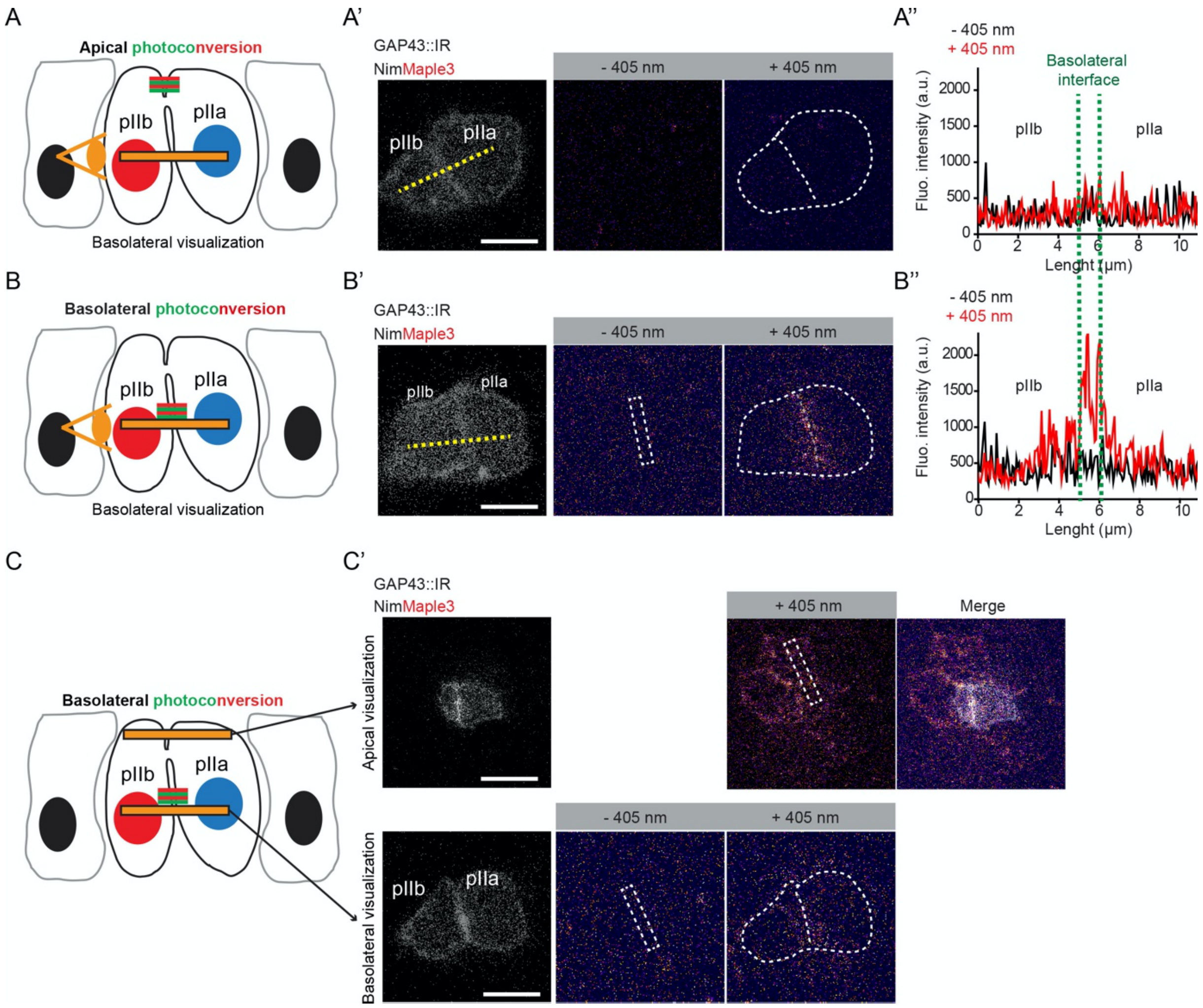

Figure S4: Spatial consequences and caveats of photoconversion of NimMaple3 at the apical and basal pIIa-pIIb interface

A-B'. Schematic representations of the apical (**A-A'**) and basolateral (**B-B'**) photoconversion assays in wild-type SOP daughter cells expressing GAP43::IR under the *neur* promoter. Orange lines show the z where the photoconverted signal is measured, at the nuclei level. **A''-B''.** Plot intensity profiles along pIIb-pIIa nuclei (dashed yellow lines in **A'** and **B'**) before (black) and after (red) apical (**A''**) or basolateral (**B''**) photoconversion. Photoconversions were performed 30 minutes after anaphase transition. **C-C'.** Schematic representations of the basolateral photoconversion assay in wild-type SOP daughter cells expressing GAP43::IR under the *neur* promoter. Orange lines show the z where the photoconverted signal is imaged, at the apical level. Photoconversions were performed 30 minutes after anaphase transition. Dashed white rectangles show basolateral photoconversion areas and dashed white lines highlight SOP daughter cells

Development • Supplementary information

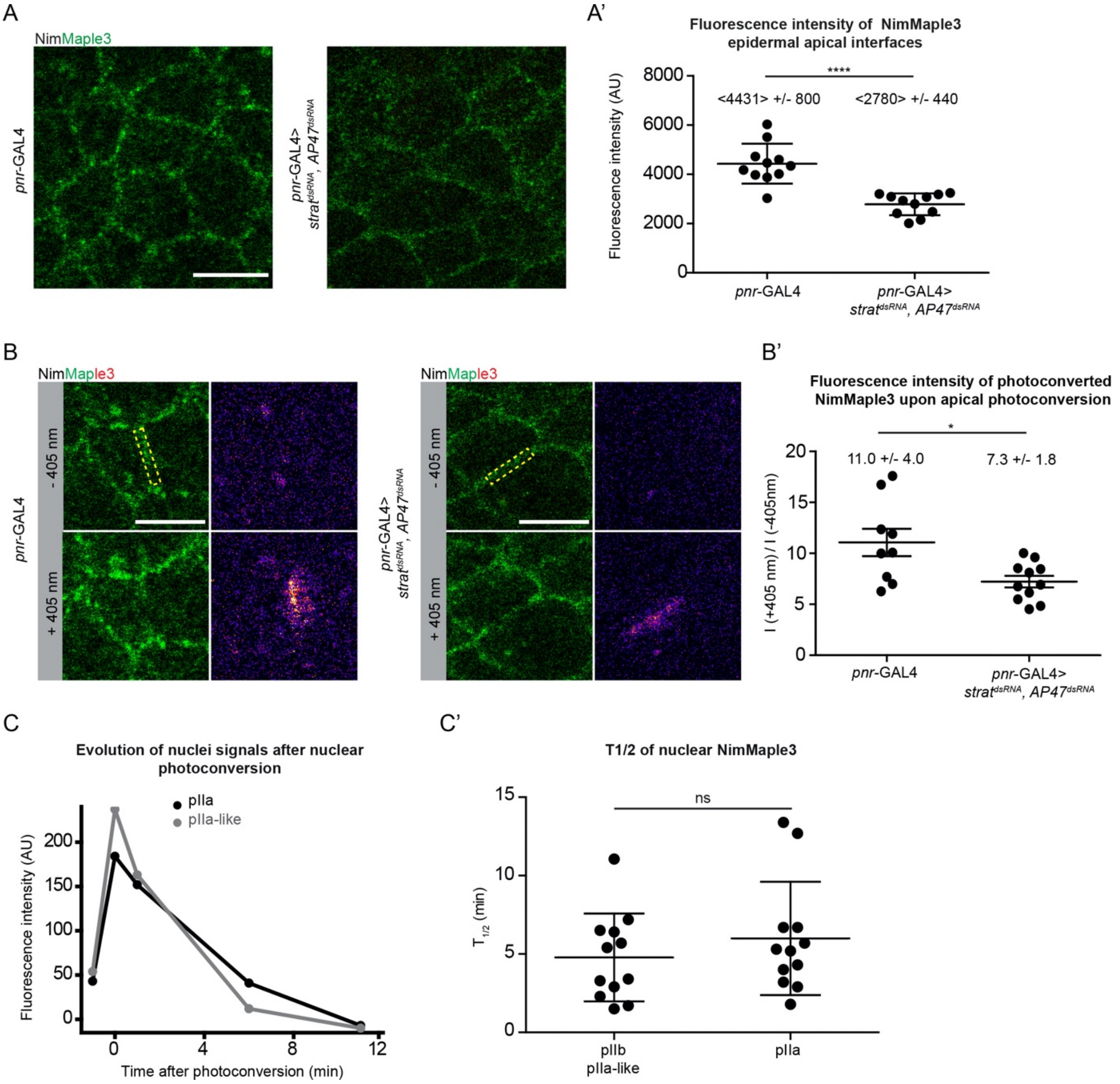

Figure S5: Relative levels of NimMaple3 signals in control versus upon loss of Strat and AP-1

A. Localization of NimMaple3 at the apical interface between epidermal cells in pupae expressing *pnr*-GAL4 or expressing *pnr*-GAL4>*strat*^{dsRNA}*, AP-47*^{dsRNA}. **A'.** Plot of fluorescence intensity of NimMaple3 at the apical interface between epidermal cells in pupae expressing *pnr*-GAL4 or *pnr*-GAL4>*strat*^{dsRNA}*, AP-47*^{dsRNA} (*****P*<0.0001). Upon loss of Strat and AP-1, the NimMaple3 signal is on average 1.59 fold lower than in control. **B.** Photoconversion of NimMaple3 present at the apical interface between epidermal cells in pupae expressing in *pnr*-GAL4 or *pnr*-GAL4>*strat*^{dsRNA}*, AP-47*^{dsRNA}. Dashed yellow ROI delineate the photoconverted area. Scale bar is 5 µm. **B'.** Plot of fluorescence intensity of the photoconverted NimMaple3, at the apical interface between epidermal cells and after apical photoconversion, in pupae expressing *pnr*-GAL4 or *pnr*-GAL4>*strat*^{dsRNA}*, AP-47*^{dsRNA} (**P*<0.05). Upon loss of Strat and

Development • Supplementary information

AP-1, the photoconverted NimMaple3 signal is on average 1.5 fold lower than in control. **C.** Example of a profile corresponding to the evolution of *pnr*-GAL4>*strat*^{dsRNA}, *AP-47*^{dsRNA} SOP daughter cells nuclei signals upon nuclear photoconversion at 30 minutes after anaphase. T0 corresponds to the nuclear photoconversion. **C'.** Plot of T1/2 of nuclear NimMaple3 in *pnr*-GAL4>*strat*^{dsRNA}, *AP-47*^{dsRNA} SOP daughter cells. (ns≥0.05).

Development • Supplementary information

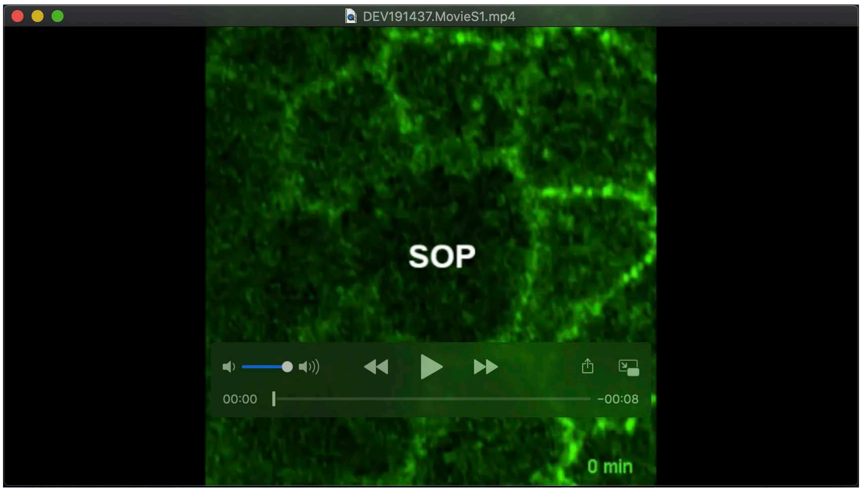

Movie 1: Dynamic of NiGFP in dividing wild-type SOP

Apical plan of a time-lapse imaging of NiGFP (green) in dividing wild-type SOP (n=10). Time is in minutes and the time 0 corresponds to the SOP anaphase onset. Asterisks show the pIIb and the pIIa cells. The apical interface between the two daughter cells is localized between the two asterisks.

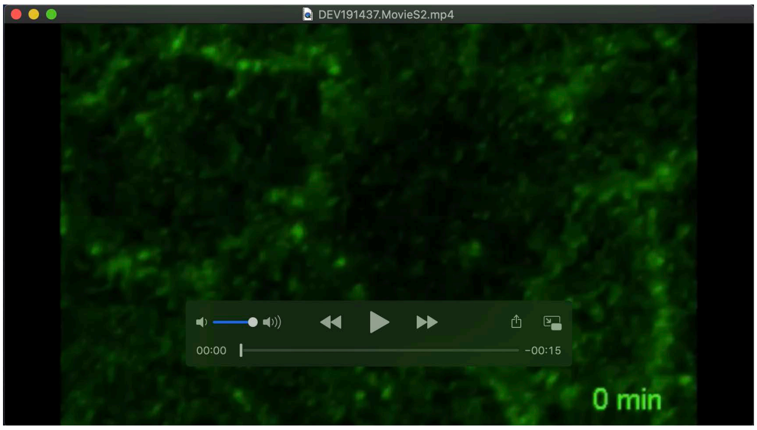

Movie 2: Dynamic of NiGFP in dividing *strat* SOP expressing *pnr*-GAL4>*AP-47*^{dsRNA}

Apical plan (Projection of the 2 most apical slices) of a time-lapse imaging of NiGFP (green) in dividing *strat* SOP expressing *pnr*-GAL4>*AP-47*^{dsRNA} (n=10). Time is in minutes and the time 0 corresponds to the SOP anaphase onset. Asterisks show the two daughter cells. The apical interface between the two daughter cells is localized between the two asterisks.

Development • Supplementary information
